# Latent profiles and associated factors of medication literacy in older adult patients with chronic diseases

**DOI:** 10.3389/fpubh.2025.1660554

**Published:** 2025-10-16

**Authors:** Zhenfan Liu, Xiaoting Yan, Jing Lu, Zhitong Wang, Chuanyu Zhou, Yan Wang, Yingying Zhong, Wei Qing

**Affiliations:** ^1^Department of Geriatrics, Deyang People's Hospital, Deyang, China; ^2^Department of Nursing, Sichuan Nursing Vocational College, Chengdu, China; ^3^Department of Nephrology, Deyang People's Hospital, Deyang, China

**Keywords:** aged, chronic disease, health literacy, self-efficacy, latent class analysis

## Abstract

**Background:**

With the global burden of chronic diseases and the acceleration of population aging, medication literacy is crucial for self-management among older adult patients. However, the potential patterns of medication literacy remain understudied, leaving us unable to clearly categorize medication literacy among older adult patients with different characteristics.

**Objective:**

The purpose of this study is to investigate the Medication Literacy of older adult patients with chronic diseases. Specifically, it aims to examine the current status of Medication Literacy in this population; to analyze distinct patterns of Medication Literacy and their relationship with chronic disease self-efficacy; and to explore the factors influencing these different patterns.

**Methods:**

A cross-sectional study was conducted using the convenience sampling method. Chronic disease patients admitted to the geriatrics department of a tertiary-level hospital in Deyang City, China were recruited between January and June 2025, with a final sample size of 316 participants. A general information questionnaire, a medication literacy scale for older adult patients with chronic diseases, and a chronic disease self-efficacy scale were used to conduct the survey. Latent profiles of medication literacy among these patients were identified using Mplus 8.3. Logistic regression was employed using SPSS23.0 to analyse the factors influencing different categories of medication literacy.

**Results:**

Finally, 316 older adult patients with chronic diseases were included. Older adult patients with chronic diseases had a total medication literacy median score 70.50 (IQR: 50.00, 89.00) and a total disease self-efficacy median score 47.00 (IQR: 38.00, 52.00). Medication literacy of older adult patients with chronic diseases can be classified into four potential categories: comprehensive deficiency type (16.8%), communication strength type (28.8%),balanced development type(29.7%), and knowledge proficiency type(24.7%). Logistic regression analysis showed that age, education, personal monthly income, and chronic disease self-efficacy were associated factors of medication literacy in older adult patients with chronic diseases (all *p* < 0.05).

**Conclusion:**

Overall, medication literacy among older adult patients with chronic diseases is at a moderate level and shows heterogeneity. Future prospective studies should test hypotheses such as: To address this, healthcare professionals should prioritize patients falling into the comprehensive-deficiency and communication-strength types, developing tailored interventions to enhance their competencies based on these distinct characteristics.

## 1 Introduction

Currently, countries worldwide are confronting challenges posed by population aging. According to data from the National Bureau of Statistics of China in 2024, the number of older adults aged 60 years and above in China reached 310 million, accounting for 22.0% of the total population (1, 2). The rapid growth of this population has made China one of the most deeply aging countries globally, with far-reaching impacts on various aspects: socioeconomic factors, medical resource allocation, and the public health system.

With the continuous development of society and economy, as well as changes in people’s lifestyles, the disease spectrum among the older adults has also changed accordingly. This has caused the incidence and prevalence of chronic non-communicable diseases, such as cardiovascular diseases, cerebrovascular diseases, respiratory diseases, and cases of diabetes mellitus, to show a continuous rising trend (3). The proportion of Chinese residents over 60 years old with at least one chronic disease is as high as 75.8%, and the burden of chronic diseases is on the rise (4). In addition, nearly 3.8 million older people in China die of chronic diseases every year, accounting for 83.4% of all deaths among older people (4). Meanwhile, the World Health Statistics Report 2021 also reports that chronic diseases accounted for seven of the top ten causes of death in 2019, and the mortality rate of chronic diseases increased from 60.8% in 2000 to 73.6% in 2019 (5). Together, these data show that the prevalence and mortality of chronic diseases are increasing globally. They have become major health problems threatening human health and are bringing a huge burden to the global economy and social development (6).

Most chronic diseases in the older adults are lifelong and incurable, requiring patients to take one or more medications for extended periods, often for the rest of their lives. Due to the decline in physical functions, the cognitive abilities of the older adults—such as memory and comprehension—also deteriorate to varying degrees. This decline makes it difficult for them to accurately understand medication-related information, such as dosage instructions and potential side effects, which in turn affects proper medication use (7, 33). The therapeutic effectiveness of medication, a key component of chronic disease treatment, depends not only on the Medication’s efficacy but also on the patient’s correct understanding of medication information and their ability to use the medication safely and appropriately (8).

Medication literacy, as an important component of health literacy in the field of medication use, mainly refers to the ability of patients to correctly obtain, understand, and evaluate Medication-related information in order to make safe and effective medication decisions (7, 9, 10). Studies have found that medication literacy can directly improve medication adherence and ensure the rational and safe use of medication in the older adults, thereby improving their quality of life (11). In addition, medication literacy is negatively correlated with frailty; the higher the medication literacy, the better patients maintain good medication habits and adhere to the treatment regimen, which reduces the incidence of frailty (12). Thus, improving medication literacy in older adult patients with chronic diseases is crucial to enhancing their health outcomes.

At this stage, relevant studies on medication literacy have been conducted both domestically and internationally. Studies have found that patients’ medication literacy is affected by many factors, including general personal information such as age, education, and type of disease, as well as social support, family care, and beliefs about taking medication (13, 14). Chronic disease self-efficacy, as an individual’s intrinsic positive psychological trait, mainly refers to the patient’s confidence and belief that they have the ability to manage the disease when faced with it. According to social cognitive theory, an individual’s self-efficacy can directly affect their psychological state. This effect enables the individual to have the confidence and ability to manage the disease, making them more willing to adopt positive health behaviors (15).

However, previous studies on medication literacy have mainly focused on current status surveys and the classification of high or low levels of medication literacy, without considering the variability among groups with different characteristics, which is key to implementing accurate interventions. At the same time, relevant investigations have primarily focused on the entire population, with few studies concentrating on medication literacy in older patients with chronic diseases and its relationship with chronic disease self-efficacy. Latent profile analysis, on the other hand, is a categorical statistical method focusing on individuals. It can accurately identify latent categories within groups, classify individuals with similar characteristics into the same category, and estimate the probability of class membership. This allows researchers to clearly understand the relationships among different types of individuals, clarify the nature and number of categories, and provide an effective basis for precise interventions (16). Therefore, this study mainly used the latent profile approach to explore the potential profiles of medication literacy in older adult patients with chronic diseases. At the same time, it analyzed the characteristics and associated factors of patients in different profiles and explored the relationship between medication literacy and disease self-efficacy across these profiles, aiming to provide a more personalized medication guidance intervention as a reference.

## 2 Methods

### 2.1 Research designs and participants

This study is a cross-sectional study. The convenience sampling method was used, and 316 patients with chronic diseases hospitalized in the geriatrics department of a tertiary hospital in Deyang City were selected from January to June 2025 as the survey subjects. The inclusion criteria for patients were as follows: (1) clinically diagnosed with at least one chronic disease; (2) age over 60 years; (3) informed consent and voluntary participation in this survey. The exclusion criteria were: (1) consciousness disorder or intellectual disability, resulting in inability to communicate normally; (2) patients in the acute stage of disease, not suitable for the survey. Sample size calculation: According to Kendall’s criterion, the sample size for regression analysis should be 10–20 times the number of independent variables. This study included 15 independent variables, resulting in a sample size of 150–300 (17). Considering a 20% non-response rate, the minimum sample size N = 15 × 10 + [(15 × 10) × 20%] = 180. A total of 330 questionnaires were collected in this study. After screening, 14 questionnaires with inconsistent responses or obvious logical errors (such as selecting the same option for all items) were completely excluded, yielding 316 valid questionnaires for final analysis. The effective questionnaire recovery rate was 95.8%. The study was approved by the Ethics Committee (No. 2022-04-010-K01).

### 2.2 Survey

#### 2.2.1 Sociodemographic and clinical characteristics of the participants

The general information questionnaire was developed by the project team members after reviewing the literature. It consisted of two parts: (1) Sociodemographic characteristics of the participants, including age, gender, place of residence, education, marital status, monthly income, number of children, and Type of medical insurance; and (2) Clinical characteristics of the participants, including number of chronic diseases, disease duration, number of medications currently used, whether patients undergo regular medical reviews, and history of adverse Medication reactions. Two clinical experts and two nursing experts were invited to evaluate the content validity of the questionnaire, with a Content Validity Index (CVI) of 0.930.

#### 2.2.2 Self-efficacy for managing chronic disease 6-item scale, SES6G

Created by Lorig (18) from Stanford University in the United States. The Chinese version of SECD6 has good internal consistency, with a Cronbach’s alpha of 0.91 (19). The scale comprises six items grouped into two subscales: Symptom Management Self-Efficacy (4 items) and Disease Common Management Self-Efficacy (2 items). Responses are recorded using a 10-point Likert scale, ranging from 1 (‘Not at all confident’) to 10 (‘Completely confident’). The total score ranges from 6 to 60 points, with higher scores indicating better self-efficacy. Cronbach’s alpha was 0.976 in this study.

#### 2.2.3 Medication literacy scale for older adult patients with chronic diseases

The scale was developed by Chinese scholar Zhao Xue (20) and demonstrates good internal consistency, with a Cronbach’s alpha coefficient of 0.958. The 23-item scale comprises four dimensions: information acquisition ability (5 items), disease knowledge base (6 items), communication and interaction ability (5 items), and critical thinking ability (7 items). Items are rated on a 5-point Likert scale (1 = impossible, 5 = completely possible). Total scores range from 23 to 115, with higher scores indicating better medication literacy. Cronbach’s alpha was 0.975 and McDonald‘s omega was0.978 in this study. Confirmatory factor analysis revealed: χ^2^/df = 4.30 (*p* < 0.001), CFI = 0.909, TLI = 0.897, RMSEA = 0.102 (95% CI: 0.096–0.109), SRMR = 0.053. We employed Multi-Group Confirmatory Factor Analysis (MG-CFA) to examine the measurement invariance of the Medication Literacy Scale across different education level groups (primary school and below vs. junior high school vs. high school and above). Although the absolute fit indices of the baseline model (configural invariance) did not reach ideal standards (χ^2^/df = 2.88, CFI = 0.888, TLI = 0.877, RMSEA = 0.097), nested model comparisons revealed that neither the metric invariance model (ΔCFI = −0.003, ΔRMSEA = 0.005) nor the scalar invariance model (ΔCFI = −0.005, ΔRMSEA = 0.003) showed a significant degradation in fit compared to their more relaxed counterparts (ΔCFI > − 0.01). This indicates that the Medication Literacy Scale possesses scalar measurement invariance among older adult patients with chronic diseases across different education levels, demonstrating identical measurement structure, factor loadings, and item intercepts. This ensures that subsequent group comparisons and the interpretation of mean differences based on scale scores are valid and reliable.

#### 2.2.4 Procedure

This study was conducted using a face-to-face questionnaire. First, the research team designed the questionnaire based on study objectives, incorporating standard informed consent procedures, background/purpose explanations, completion guidelines, and confidentiality assurances. The instrument was refined following a pilot survey to establish the final version. Subsequently, trained investigators administered the survey. Participants self-completed questionnaires after providing informed consent. For illiterate participants, investigators verbally recorded responses based on their answers after thorough explanation of the content. Finally, all questionnaires were distributed and collected anonymously on-site. Data coding and access were restricted to the research team.

### 2.3 Statistical analysis

Latent profile analysis (LPA) was conducted in Mplus 8.3. Model fit indices included: AIC, BIC, aBIC (lower = better), Entropy (0–1; higher = better classification,an Entropy value ≥ 0.80 suggests approximately 90% accuracy in class assignment), LMR-LRT and BLRT (*p* < 0.05 favored k-class over (k-1)-class models) (21). Analyses used SPSS 23.0. Mean ± SD (normally distributed) Median (P25, P75) (non-normal) *n* (%) (categorical). Group comparisons: Kruskal-Wallis (continuous), χ^2^/Fisher’s exact (categorical). Predictors of medication literacy profiles were analyzed via ordinal logistic regression. A *p*-value of < 0.05 indicated statistical significance.

## 3 Results

### 3.1 General characteristics

Among the 316 included patients, 176 (55.7%) were male and 140 (44.3%) were female, with a mean age of 74.94 years (SD = 8.89). A total of 77.8% were married, and 74.1% had more than one chronic disease. Among the 14 excluded patients, 9 (64.3%) were male and 5 (35.7%) were female, with a mean age of 74.07 years (SD = 5.01). A total of 64.3% were married, and 64.3% had more than one chronic disease. Sensitivity analysis was conducted by comparing the demographic and clinical characteristics between the included participants (*n* = 316) and the excluded participants (*n* = 14). The results showed no significant differences between the two groups in terms of gender, age, education level, or number of chronic diseases (all *p* > 0.05), indicating that the exclusion of cases did not introduce significant bias. We further conducted a best-worst case scenario analysis to test the impact of extreme assumptions regarding the missing data. Under the worst-case scenario (assuming all 14 excluded participants belonged to the lowest medication literacy profile), and the best-case scenario (assuming all belonged to the highest profile), the results of the ordinal logistic regression model were re-examined. The significance and direction of the associations for the key predictors (age, education, monthly income, and self-efficacy) remained unchanged in both scenarios, confirming the robustness of our primary findings even under highly conservative assumptions. Additional characteristics are presented in Table 1.

**Table 1:** Participants’ n demographic and clinical characteristics.

Characteristics	Groups		Test statistic	*p*-value
	Included participants (*n* = 316)	Excluded participants (*n* = 14)		
Gender			0.402	0.526
Male	176 (55.7%)	9 (64.3%)		
Female	140 (44.3%)	5 (35.7%)		
Place of residence			0.070	0.791
Rural	124 (39.2%)	5 (35.7%)		
Urban	192 (60.8%)	9 (64.3%)		
Marital status			1.404	0.236
Married	246 (77.8%)	9 (64.3%)		
Divorced or widowed	70 (22.2%)	5 (35.7%)		
Number of children(number)			1.621	0.445
≤ 1	118 (37.3%)	7 (50.0%)		
2	92 (29.1%)	2 (14.3%)		
≥ 3	106 (33.5%)	5 (35.7%)		
Education			4.090	0.129
Primary and below	177 (56.0%)	10 (71.4%)		
Junior high school	67 (21.2%)	4 (28.6%)		
High school and above	72 (22.8%)	0 (0.0%)		
Personal monthly income (RNB, yuan)			1.080	0.782
≤1,000	109 (34.5%)	4 (28.6%)		
1,001 ~ 3,000	82 (25.9%)	5 (35.7%)		
3,001 ~ 5,000	5617.7% ()	3 (21.4%)		
≥5,001	69 (21.8%)	2 (14.3%)		
Type of medical insurance			1.245	0.265
Employee medical insurance	161 (50.9%)	5 (35.7%)		
Resident medical insurance	155 (49.1%)	9 (64.3%)		
Age (years)			5.441	0.142
60 ~ 64	53 (16.8%)	0 (0.0%)		
65 ~ 74	103 (32.6%)	8 (57.1%)		
75 ~ 89	145 (45.9%)	6 (42.9%)		
≥90	15 (4.7%)	0 (0.0%)		
Number of chronic diseases (number)			2.947	0.229
1	82 (25.9%)	5 (35.7%)		
2 ~ 4	181 (57.3%)	9 (64.3%)		
>5	53 (16.8%)	0 (0.0%)		
Disease duration (years)			3.170	0.530
<1	29 (9.2%)	1 (7.1%)		
1 ~ 5	80 (25.3%)	4 (28.6%)		
6 ~ 10	84 (26.6%)	6 (42.9%)		
11 ~ 20	88 (27.8%)	3 (21.4%)		
>20	35 (11.1%)	0 (0.0%)		
Number of medications used (number)			1.761	0.414
≤1	78 (24.7%)	4 (28.6%)		
2 ~ 4	122 ()38.6%	3 (21.4%)		
≥5	116 (36.7%)	7 (50.0%)		
Whether regular checkups are received			2.022	0.155
Yes	174 (55.1%)	5 (35.7%)		
No	142 (44.9%)	9 (64.3%)		
Any previous adverse medication reactions			0.075	0.785
Yes	54 (17.1%)	2 (14.3%)		
No	262 (82.9%)	12 (85.7%)		

### 3.2 Medication literacy, disease self-efficacy scores of older adult patients with chronic diseases

The results of this study showed that the total score of Medication Literacy and Disease Self-Efficacy of Older adult patients with Chronic Diseases was 70.50 (50.00, 89.00) and the total score of Disease Self-Efficacy was 47.00 (38.00, 52.00), where the scores of each of the dimensions are presented in Table 2.

**Table 2:** Scores of medication literacy and disease self-efficacy in older adult patients with chronic diseases.

Items	Median score [25, 75%]	Score rate
Total medication literacy score	70.5 (50.00, 89.00)	61.3%
Information acquisition ability	11.50 (8.00, 19.00)	46.0%
Medication knowledge reserve	19.00 (13.00, 25.00)	63.3%
Communication and interaction ability	15.00 (12.00, 20.00)	60.0%
Critical ability	22.00 (15.00, 27.00)	62.9%
Total disease self-efficacy	47.00 (38.00, 52.00)	78.3%
Symptom management self-efficacy	31.00 (25.00, 34.75)	77.5%
Disease comorbidity management self-efficacy	16.00 (13.00, 18.00)	80.0%

### 3.3 This section presents the results of the analysis of latent profiles of medication literacy scale for older adult patients with chronic diseases

In this study, item scores of medication literacy among older adult chronic disease patients were used as manifest indicators. Initial models with 1 to 5 latent profiles were fitted, and the fit indices for each model are presented in Table 3. As the number of latent profiles increased, the AIC, BIC, and aBIC values demonstrated monotonic decreases. The entropy values for all five-class solutions exceeded 0.800, and the Bootstrap Likelihood Ratio Test (BLRT) results were statistically significant across all latent profiles. However, the Lo-Mendell-Rubin adjusted likelihood ratio test (LMR) for the five-class model was not statistically significant (*p* > 0.05), suggesting that adding more classes might alter model stability. Based on comprehensive evaluation, the four-class model was identified as the optimal solution, consequently retaining four distinct profiles (C1, C2, C3, C4).

**Table 3:** Latent profile of medication literacy in older adult patients with chronic diseases and model fit indices for each model.

Models	*AIC*	*BIC*	*aBIC*	*LMR*	*BLRT-p*	*Entropy*	Profile scale
1	24093.519	24266.283	24120.382	–	–	–	–
2	19511.851	19774.753	19552.731	<0.001	<0.001	0.982	0.484/0.516
3	18422.951	18775.991	18477.847	0.0034	<0.001	0.971	0.307/0.326/0.367
4	17791.123	18234.301	17860.035	0.0287	<0.001	0.962	0.168/0.288/0.297/ 0.247
5	17504.193	18037.509	17587.121	0.0745	<0.001	0.978	0.165/0.269/0.196/0.234/0.136

To evaluate the accuracy of the latent profile classification, we examined the posterior classification metrics of the model. As shown in Table 4, the average posterior probabilities for the four latent profiles were 0.988, 0.990, 0.976, and 0.971, all well above the acceptable threshold of 0.90. Furthermore, the diagonal values of the classification probability matrix were all greater than 0.90, while the off-diagonal values were consistently low (all < 0.05). In addition, the average variance extracted (AVE) exceeded 0.6, and the composite reliability (CR) surpassed 0.9, collectively indicating high within-class homogeneity. These results demonstrate that the model’s classification of individuals is highly accurate and reliable, with a very low probability of misclassification.

**Table 4:** Accuracy indicators for latent profile classification.

Latent profile	Average posterior probability	Classification probability (C1)	Classification probability (C2)	Classification probability (C3)	Classification probability (C4)
C1	0.988	0.987	0.013	0.000	0.000
C2	0.990	0.007	0.981	0.012	0.000
C3	0.976	0.000	0.003	0.973	0.024
C4	0.971	0.000	0.000	0.015	0.985

### 3.4 Characteristics and naming of potential profiles of medication literacy in older adult patients with chronic diseases

**Figure 1:**
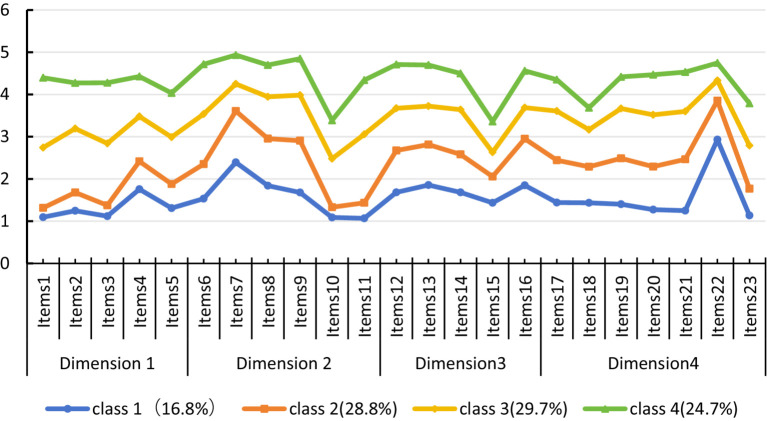
Distribution of the four potential characteristics of medication literacy among the older adult.

Based on the conceptual model of medication literacy proposed by Pantuzza et al. (7), the four identified types of medication literacy among older adult patients were named according to their performance across measurement dimensions (see Figure 1): C1: Comprehensive-Deficit Type (*n* = 53, 16.8%): This profile demonstrated significantly lower scores across all dimensions, with particularly notable deficiencies in functional literacy (e.g., information acquisition and comprehension) and critical literacy (decision-making abilities). C2: Communication-Advantage Type (*n* = 91, 28.8%): This group showed low to moderate overall performance but exhibited relative strength in communicative literacy (e.g., patient-provider interaction and medication communication), while functional and critical literacy remained low. C3: Balanced-Development Type (*n* = 94, 29.7%): This profile displayed well-balanced capabilities across all dimensions, reflecting comprehensive literacy skills with an overall intermediate level of proficiency. C4: Knowledge-Proficient Type (*n* = 78, 24.7%): This group achieved the highest scores across all dimensions, with particularly outstanding performance in critical literacy (e.g., application of disease knowledge and decision-making), coupled with high functional and interactive literacy.

### 3.5 Univariate analysis of potential medication literacy profiles of older adult patients with chronic diseases

The results of univariate analysis showed that the differences in the distribution of medication literacy profiles among older adult patients with chronic diseases residing in different places, marital status, number of children, education, monthly income, type of medical insurance, age, Whether regular checkups are received, and self-efficacy in managing their diseases were statistically significant (*p* < 0.05). Details are provided in Table 5.

**Table 5:** Univariate analysis of medication literacy profiles in older adult patients with chronic diseases.

Item	Class 1	Class 2	Class 3	Class 4	Test statistic	*p*-value
Gender					6.407^a^	0.093
Male	26 (49.1%)	45 (49.5%)	62 (66.0%)	43 (55.1%)		
Female	27 (50.9%)	46 (50.5%)	32 (34.0%)	35 (44.9%)		
Place of residence					82.757^a^	0.000
Rural	36 (67.9%)	58 (63.7%)	25 (26.6%)	5 (6.4%)		
Urban	17 (32.1%)	33 (36.3%)	69 (73.4%)	73 (93.6%)		
Marital status					24.691^a^	0.000
Married	29 (54.7%)	68 (74.7%)	82 (87.2%)	67 (85.9%)		
Divorced or widowed	24 (45.3%)	23 (25.3%)	12 (12.8%)	11 (14.1%)		
Number of children (number)					38.741^a^	0.000
≤ 1	12 (22.6)	24 (26.4)	37 (39.4)	45 (57.7)		
2	10 (18.9)	29 (31.8)	34 (36.2)	19 (24.4)		
≥ 3	31 (58.5)	38 (41.8)	23 (24.4)	14 (17.9)		
Education					126.231	0.000
Primary and below	46 (86.8%)	73 (80.2%)	48 (51.1%)	10 (12.8%)		
Junior high school	4 (7.5%)	14 (15.4%)	28 (29.8%)	21 (26.9%)		
High school and above	3 (5.7%)	4 (4.4%)	18 (19.1%)	47 (60.3%)		
Personal monthly income(RNB,yuan)					130.860	0.000
≤1,000	35 (66.0%)	49 (53.8%)	23 (24.5%)	2 (2.6%)		
1,001 ~ 3,000	14 (26.4%)	25 (27.5%)	28 (29.8%)	15 (19.2%)		
3,001 ~ 5,000	4 (7.6%)	10 (11.0%)	24 (25.5%)	18 (23.1%)		
≥5,001	0 (0.0%)	7 (7.7%)	19 (20.2%)	43 (55.1%)		
Type of medical insurance					90.760^a^	0.000
Employee medical insurance	9 (17.0%)	25 (27.5%)	59 (62.8%)	68 (87.2%)		
Resident medical insurance	44 (83.0%)	66 (72.5%)	35 (37.2%)	10 (12.8%)		
Age (years)					50.534	0.000
60 ~ 64	2 (3.8%)	4 (4.4%)	25 (26.6%)	22 (28.2%)		
65 ~ 74	9 (17.0%)	34 (37.4%)	30 (31.9%)	30 (38.5%)		
75 ~ 89	36 (67.9%)	48 (52.7%)	36 (38.3%)	25 (32.1%)		
≥90	6 (11.3%)	5 (5.5%)	3 (3.2%)	1 (1.3%)		
Number of chronic diseases (number)					7.671^a^	0.263
1	18 (34.0%)	22 (24.2%)	25 (26.6%)	17 (21.8%)		
2 ~ 4	24 (45.3%)	52 (57.1%)	52 (55.3%)	53 (67.9%)		
>5	11 (20.7%)	17 (18.7%)	17 (18.1%)	8 (10.3%)		
Disease duration (years)					11.013	0.515
<1	6 (11.3%)	11 (12.0%)	8 (8.5%)	4 (5.1%)		
1 ~ 5	18 (34.0%)	20 (22.0%)	19 (20.2%)	23 (29.5%)		
6 ~ 10	12 (22.6%)	28 (30.8%)	22 (23.4%)	22 (28.2%)		
11 ~ 20	11 (20.8%)	22 (24.2%)	33 (35.1%)	22 (28.2%)		
>20	6 (11.3%)	10 (11.0%)	12 (12.8%)	7 (9.0%)		
Number of medications used (number)					4.711^a^	0.581
≤1	11 (20.8%)	22 (24.2%)	20 (21.2%)	25 (32.1%)		
2 4~	20 (37.7%)	34 (37.4%)	37 (39.4%)	31 (39.7%)		
≥5	22 (41.5%)	35 (38.4%)	37 (39.4%)	22 (28.2%)		
Whether regular checkups are received					27.058^a^	0.000
Yes	19 (35.8%)	43 (47.3%)	51 (54.3%)	61 (78.2%)		
No	34 (64.2%)	48 (52.7%)	43 (45.7%)	17 (21.8%)		
Any previous adverse medication reactions					7.611^a^	0.055
Yes	9 (17.0%)	10 (11.0%)	24 (25.5%)	11 (14.1%)		
No	44 (83.0%)	81 (89.0%)	70 (74.5%)	67 (85.9%)		
Chronic disease self efficacy	37.00 (30.00, 40.00)	54.00 (49.00, 57.00)	78.00 (73.00, 85.00)	99.00 (94.00, 106.25)	68.622^b^	0.000

Statistical methods: a, χ^2^ test (Chi-square test); b, Kruskal-Wallis H test.

### 3.6 Ordered multicategorical logistic regression of potential profiles of medication literacy in older adult patients with chronic diseases

Using variables that showed statistical significance in the univariate analysis as independent variables (Place of residence: rural = 1, urban = 2; Marital status: married = 1, divorced or widowed = 2; Number of children: ≤1 = 1, 2 = 2, ≥3 = 3; Education level: primary school and below = 1, junior high school = 2, high school and above = 3; Personal monthly income (yuan): ≤1,000 = 1, 1,001–3,000 = 2, 3,001–5,000 = 3, ≥5,001 = 4; Health insurance type: employee insurance = 1, resident insurance = 2; Age (years): 60–64 = 1, 65–74 = 2, 75–89 = 3, ≥90 = 4; Regular follow-up: yes = 1, no = 2; Disease self-efficacy was entered as the actual score), and the latent profiles of medication literacy in older adult patients with chronic diseases as the dependent variable (Comprehensive Deficiency Type = 1, Communication Strength Type = 2, Balanced Development Type = 3, Knowledge Proficiency Type = 4), an ordered multinomial logistic regression analysis was performed using the Comprehensive Deficiency Type as the reference group. The mean scores of each dimension across the four profiles showed a sequentially increasing trend, and the test of parallel lines (χ^2^ = 40.552, *p* = 0.095) indicated that the ordered multinomial logistic regression model could be adopted; the Pearson goodness-of-fit test (*χ*^2^ = 611.576, *p* > 0.05) indicated good model fit, and the overall fit test showed that the regression model was statistically significant (*p* < 0.001). The results revealed that age, education level, personal monthly income, and disease self-efficacy significantly predicted the health literacy of older adult patients, as shown in Table 6.

**Table 6:** Ordered multicategorical logistic regression of potential profiles of medication literacy in older adult patients with chronic diseases.

Item	*β*	*SE*	*p*	OR (95% CI)
Total disease self-efficacy score	0.067	0.014	0.000	1.069 (0.039, 0.095)
Age (reference: ≥90 years)				
60 ~ 64	2.262	0.677	0.001	9.602 (0.935, 3.589)
65 ~ 74	2.289	0.636	0.000	9.865 (1.042, 3.536)
75 ~ 89	1.420	0.581	0.015	4.137 (0.280, 2.559)
Education(reference: High school and above)				
Primary and below	−1.532	0.388	0.000	0.216 (−2.294, −0.771)
Junior high school	−0.797	0.383	0.038	0.451 (−1.549, −0.046)
Place of residence (reference: urban)				
Rural	−0.591	0.325	0.069	0.554 (−1.228, 0.046)
Marital status (reference: Divorced or widowed)				
Married	−0.099	0.300	0.740	0.906 (−0.687, 0.488)
Number of children (reference: ≥3)				
≤ 1	0.121	0.326	0.710	1.129 (−0.518, 0.760)
2	0.485	0.308	0.115	1.624 (−0.118, 1.088)
Personal monthly income (RNB, yuan) (reference: ≥5,001)				
≤ 1,000	−1.907	0.506	0.000	0.149 (−2.898, −0.915)
1,001 ~ 3,000	−1.363	0.437	0.002	0.256 (−2.219~, −0.506)
3,001 ~ 5,000	−0.895	0.399	0.025	0.409 (−1.677, −0.113)
Type of medical insurance(reference: Resident medical insurance)				
Employee medical insurance	0.367	0.353	0.299	1.443 (−0.325, 1.059)
Whether regular checkups are received (reference: no)				
Yes	0.118	0.248	0.633	1.125 (−0.367, 0.604)

## 4 Discussion

### 4.1 Medication literacy of older adults with chronic diseases is at an intermediate level

The results of this study indicate that the total medication literacy score among older adult patients with chronic diseases was 70.50 (50.00, 89.00), with a scoring rate of 61.3%, which is at a moderate level and warrants further improvement. This finding is consistent with the interview results reported by Rahman et al. (22). Regarding patients with chronic conditions. Due to aging, the vision, hearing, and cognitive abilities of older adults decline, making it difficult for them to receive, understand, and remember Medication information. At the same time, incorrect medication attitudes and behaviors have led some older adults to not take standardized medication as prescribed, which further hinders the improvement of their medication literacy (11). The information acquisition ability dimension scored the lowest at 46.0%. This may be because, due to their limited knowledge and educational background, older adults have difficulties accurately interpreting and understanding complex Medication instructions and medical terminology, thus hindering their ability to effectively acquire medication-related information. In addition, older adults have relatively limited access to Medication information channels, mostly relying on guidance from medical staff. Their ability to apply information technology is relatively weak, which limits their effective use of the Internet and other platforms to obtain diversified Medication information (23).

### 4.2 Potential profile classification of medication literacy among older adult patients with chronic diseases

Findings from this study reveal significant heterogeneity in medication literacy among older adult patients with chronic diseases, which can be categorized into four distinct profiles: comprehensive deficiency type (16.8%), communication strength type (28.8%), balanced development type (29.7%), and knowledge proficiency type (24.7%).

The comprehensive deficiency type scored the lowest across all dimensions, accounting for 16.8% of the sample. This profile is predominantly characterized by individuals residing in rural areas, having an elementary school education or below, a monthly income ≤ ¥1,000, and being enrolled in resident health insurance. Most patients in this category live in rural settings with relatively low educational attainment and weak information acquisition skills, making it difficult for them to understand complex medication-related information, thus resulting in lower medication literacy. For these patients, healthcare providers should adopt diversified medication guidance strategies, such as using simple, humorous, and easy-to-understand language to explain complex medication knowledge; organizing drug information into large-print, illustrated medication lists and pill organizers based on patient needs to facilitate understanding for both patients and their families; and fully leveraging the supervisory role of family members through repeated verification and assessment of the patient’s medication knowledge and skills to enhance their overall medication literacy.

The communication strength type had relatively low scores across most dimensions but scored highest in the communicative interaction dimension, comprising 28.8% of the sample. This group is primarily characterized by being married, having an elementary school education or below, having 2–4 chronic diseases, and no history of adverse drug reactions. Although their low educational level makes it challenging to grasp medication knowledge, resulting in overall lower medication literacy, their multiple chronic conditions and high marriage rate enable them to acquire knowledge with family support and discuss medication matters collectively, leading to better performance in communicative interaction. This suggests that healthcare providers should capitalize on these patients’ communication strengths by recommending reliable medication management platforms, teaching them how to search for and discern medication information, and strengthening their mastery of medication knowledge.

The balanced development type showed relatively balanced, moderate scores across all dimensions, accounting for 29.7% of participants. This profile is mainly composed of male urban residents who are married and covered by employee health insurance. These patients generally benefit from relatively better healthcare access and living conditions, which contribute to their medication literacy, though there remains room for further improvement. For this group, targeted health education initiatives—such as regular medication knowledge lectures and consulting sessions—should be implemented to provide personalized medication guidance. Additionally, they should be encouraged to set self-monitoring goals for medication use, integrating knowledge with action to enhance their medication knowledge, attitudes, and behaviors (32).

The knowledge proficiency type scored the highest across all dimensions, constituting 24.7% of the sample. This profile is characterized by urban residence, high school education or above, employee health insurance, and a monthly income ≥ ¥5,001. These patients not only possess relatively high educational levels, enabling them to effectively understand and master medication-related knowledge and information, but also have sufficient income to access high-quality healthcare services and medication resources, allowing them to adhere to medical advice and use medications appropriately. Healthcare providers should affirm and support the high level of medication literacy in these patients, encouraging them to maintain their good medication habits and behaviors. Furthermore, these individuals can be trained as “Medication Health Ambassadors” and encouraged to participate in peer education, sharing their medication management experiences to improve medication literacy among more older adult patients, thereby reducing the incidence of adverse health outcomes.

### 4.3 Potential profiling associates on medication literacy in older adult patients with chronic diseases

#### 4.3.1 Age

The current study found that age was an associated factor in the potential category of medication literacy among older patients with chronic diseases, with older patients having a lower probability of belonging to the “knowledge proficiency” group. Multiple studies using diverse medication literacy assessment tools consistently demonstrate an inverse relationship between age and medication literacy levels (24). This may occur because aging naturally diminishes physiological and cognitive functions among older adult patients, while medication-related knowledge is inherently complex and medically specialized with numerous technical terms. Consequently, older adult patients often struggle to master medication knowledge and skills, lacking sufficient willingness or energy to engage with medication information. Additionally, age-related visual impairment hinders their ability to read small-font medication labels containing substantial information, further obstructing comprehension of pharmaceutical knowledge.

Future prospective studies should test hypotheses such as: healthcare professionals should prioritize older adult patients’ medication literacy levels by implementing targeted guidance strategies for complex medication information: (1) utilizing cartoons and visually engaging formats to attract attention; (2) developing easily comprehensible medication science popularization materials; (3) strengthening family education to enhance medication knowledge within households, enabling families to effectively supervise older adult patients’ medication behaviors and thereby improve Medication Literacy levels (25, 26).

#### 4.3.2 Education

The current study found that educational attainment significantly associates medication literacy class membership among older adult patients with chronic diseases. Higher education levels increase the probability of belonging to the “knowledge proficiency” group,a finding consistent with the results reported by Plaza et al. (24, 27). This phenomenon may be attributed to two key mechanisms among more educated older adult patients: (1) their enhanced comprehension abilities facilitate active engagement with complex medical knowledge, including deeper disease understanding and greater willingness to modify medication behaviors for treatment adherence; (2) their increased receptiveness to new information enables effective utilization of diverse digital channels for acquiring medication knowledge, allowing for self-evaluation of medication practices in our internet-enabled era (28, 29). Consequently, these factors collectively enable highly educated older adult patients to demonstrate superior Medication Literacy. Future prospective studies should test hypotheses such as: healthcare providers must therefore incorporate educational stratification when designing medication literacy interventions for older adult chronic disease patients by: (1) replacing homogeneous medication guidance with tiered approaches categorized by educational attainment; (2) implementing distinct intervention modalities—visual/tactile tools for illiterate/primary-educated patients, text-based+digital resources for middle school graduates, and collaborative decision-making platforms for college-educated seniors; and (3) developing targeted support systems to bridge educational disparities, thereby optimizing Medication Literacy holistically across diverse geriatric populations.

#### 4.3.3 Personal monthly income

The current study identified personal monthly income as a significant factor associated medication literacy classification among older adult chronic disease patients. Higher-income patients demonstrated greater probability of belonging to the “knowledge proficiency” group. This association occurs because financially stable patients exhibit stronger treatment motivation, enabling them to allocate greater financial and cognitive resources toward medication information acquisition. They proactively learn pharmacological knowledge to make informed medication decisions. Additionally, patients with superior economic status access broader therapeutic channels, facilitating exposure to comprehensive medication information that further enhances medication literacy. Future prospective studies should test hypotheses such as: healthcare providers should therefore prioritize economically disadvantaged older adult patients by: (1) optimizing medication regimens to reduce financial burdens; (2) intensifying health education to emphasize treatment adherence; and (3) preventing adverse outcomes from medication discontinuation or irregular usage through behavioral regulation interventions (30).

#### 4.3.4 Chronic disease self efficacy

Logistic regression analyses revealed disease self-efficacy as a significant predictor of medication literacy classification among older adult chronic disease patients. Higher self-efficacy substantially increased the probability of belonging to the “knowledge proficiency” group (34). This association emerges because self-efficacy facilitates disease self-management behaviors - patients with elevated self-efficacy actively engage with health concerns, diligently acquire medication knowledge, and meticulously adhere to clinical instructions through full therapeutic engagement (35). This proactive learning process simultaneously enhances medication literacy and strengthens self-management capabilities, enabling more older adult chronic disease patients to achieve comprehensive medication literacy proficiency. Future prospective studies should test hypotheses such as: healthcare professionals should prioritize patients with low disease self-efficacy by implementing targeted interventions that mobilize external support systems. Clinicians must develop personalized medication guidance plans aligned with individual patient needs while intensifying medication knowledge dissemination to enhance comprehension of medication protocols, thereby building medication confidence and self-identity (31). Simultaneously, families should provide both emotional reinforcement and structured coaching to cultivate patients’ self-management confidence, ultimately enhancing self-efficacy and medication literacy proficiency.

## 5 Limitations

This study has several limitations due to constraints in time and resources. (1) The study was conducted as a single-center survey at only one Class A tertiary hospital, and all participants were recruited from tertiary hospital settings. Compared to older adult patients in primary care institutions, these participants may possess a higher level of health awareness, which limits the generalizability of the findings to the broader older adults population. Future research should strengthen multi-center collaborative investigations to enhance the generalizability and applicability of the results. (2) Although the sample size was adequate, the use of convenience sampling may introduce selection bias, potentially compromising the representativeness of the sample. Future studies could employ random sampling or sensitivity analyses to further control for bias and improve the external validity of the findings. (3) The medication literacy scale used in this study was developed within the Chinese context and has not undergone international cultural adaptation validation; consequently, the results may not be applicable to older adults populations in other cultural backgrounds. Future research could involve cross-cultural studies to improve cultural adaptation. (4) As a cross-sectional investigation, this study can only analyze associations between medication literacy and associated factors but cannot establish causal relationships between variables, resulting in limited strength of causal inference. Additionally, it assessed medication literacy types at only a single time point without dynamic follow-up, making it impossible to determine whether medication literacy changes with disease progression or interventions. Future research should incorporate longitudinal observations to track changes in patients’ medication literacy at different time points. (5) Owing to constraints of time and resources, this study only proposed targeted intervention directions without fully discussing the cost-effectiveness of these interventions, such as the cost differences and cost–benefit ratios of interventions tailored to patients with varying communication abilities and information acquisition capabilities. Future studies should incorporate health economic indicators to develop economically feasible intervention plans for patients with different characteristics. (6) Despite comparisons of demographic and clinical characteristics between included and excluded cases, our primary complete-case analysis remains reliant on the assumption of data being missing completely at random (MCAR). This is a strong assumption that may not hold in observational studies of older adults populations, where missingness could be related to unmeasured factors, such as subtle cognitive decline not captured by our exclusion criteria. Although our best–worst case scenario analysis supported the robustness of the main findings, residual selection bias due to data not missing at random (MNAR) cannot be entirely ruled out. For instance, if patients with the lowest medication literacy were systematically more likely to provide incomplete data, the prevalence of the ‘comprehensive deficiency type’ might be underestimated. Future research should employ strategies to minimize this bias, such as collecting proxy reports (e.g., from caregivers) on medication literacy or implementing longitudinal designs to better understand patterns of missingness.

## 6 Conclusion

Latent profile analysis classified the medication literacy of older adult chronic disease patients into four distinct profiles: comprehensive deficiency type, communication strength type, balanced development type, and knowledge proficiency type, with significant intergroup heterogeneity observed across demographic and clinical variables, including age, personal monthly income, educational attainment, and disease self-efficacy. Future prospective studies should test hypotheses such as: healthcare professionals should prioritize patients exhibiting comprehensive deficiency or communication strength profiles and develop tailored interventions based on these differentiated patient typologies to enhance medication literacy competencies, thereby optimizing therapeutic outcomes and minimizing adverse health consequences.

## Data Availability

The raw data supporting the conclusions of this article will be made available by the authors, without undue reservation.
